# Ecological system theory and school-age obesity in Thailand: a participation action research for implications to practice

**DOI:** 10.1017/S1463423624000501

**Published:** 2024-10-31

**Authors:** Pennapa R. Suwannawong, Naruemon Auemaneekul, Arpaporn Powwattana, Rewadee Chongsuwat

**Affiliations:** 1 Department of Public Health Nursing, Faculty of Public Health, Mahidol University, Bangkok, Thailand; 2 Department of Nutrition, Faculty of Public Health, Mahidol University, Bangkok, Thailand

**Keywords:** children, EST, family practice, obesity, PAR, prevention

## Abstract

**Objective:**

Developing an appropriate context-based school-age obesity prevention programme, understanding the root causes of obesity in real-life situations is vital. The objectives of this study were to explore the risk factors of school-age obesity based on Ecological System Theory (EST) and develop mutual problem-solving guidelines for school-age obesity prevention.

**Methods:**

Participation Action Research (PAR) was used as the study design. The data collection employed focus group discussions, in-depth interviews, participant’s observations, together with the procedures of Appreciation, Influence, and Control (AIC) with 55 school key informants.

**Results:**

Risk factors supported by EST at all level included high-calorie intake; sedentary lifestyles; perceptions of ‘Chubby are cute’; indulgent parenting, including limited exercise area in school. PAR process guarantees the sustained context-based prevention guidelines.

**Conclusions:**

The results could be used as a policy-driven for school-based participation and environmental support in order to promote health-promoting school.

## Introduction

Recognized as a serious public health concern in many countries worldwide, the prevalence and severity of the problem of children with obesity have risen dramatically over the past few decades (CDC, 2022). Thailand is currently faced with obesity in school-age children. The numbers of students with overweight and obese conditions are on the rise at startling rates from year to year (DOH, 2019). Empirical evidence demonstrates that school-age children with obesity are a significant health issue, because obesity during the school years strongly leads to a variety of chronic diseases in adulthood with consistently significant risk factors for non-communicable chronic diseases (NCDs) (Rerksuppaphol and Rerksuppaphol 2015; Ochoa and Berge, 2017). Children and adolescents with obesity are five times more likely to become adults with obesity than other children (Simmonds *et al*., 2016). Thus, school-age children should be given top priority in the primary prevention of childhood obesity.

Although previous research clearly indicates that the causes of childhood obesity in school-age children involve child-specific behavioural factors, surrounding environmental factors such as family and school environments also have tremendous influence over moulding children’s health-related behaviour, which is consistent with the illustrates of Ecological Systems Theory (EST) on how children’s environments affect them in terms or reciprocity between individuals and the environment (Bronfenbrenner, 1977; Feeg *et al*., 2014). According to the literature reviews on EST and childhood obesity, families and parents are the most influential environmental factors for children. Such factors include family perceptions (Syrad *et al*., 2015), modelling (Chai *et al*., 2019) and parenting practices (Jago *et al*., 2011; Lloyd *et al*., 2014), followed by schools as the setting where children spend most of their time and are influenced by such things as health policy and curricula, school food environment, physical activity, and exercise (Majid *et al*., 2015). This study emphasized the participation of all stakeholders including schools, students, and families for the purpose of studying the obesity phenomenon.

Literature review showed that preventing obesity in children usually focuses on separately modifying family and school environmental conditions without paying attention to their connection and participation resulting in neither continuation nor sustainability. Literature reviews have highlighted that participation from family and school could help combat childhood obesity and reduce limitations regarding sustainability (Zhou and Cheah, 2015; Phaitrakoon *et al*., 2014).

The Participation Action Research (PAR) approach can enhance participation among students, families, and schools in a way that is useful for mutual situation analysis with the aim of future mutual practice. PAR is expected to increase a sense of belonging from the initial starting point and eventually create sustainability for mutual problem-solving among participants.

To ensure optimal uptake of future mutual practice, this study focused on analysing a mutual situation analysis of the phenomenon to gain understanding of the factors related to school-age children with obesity by employing a descriptive qualitative approach and PAR. The activities encompassed two clearly defined objectives: (1) to explore the risk factors of school-age obesity based on Ecological System Theory (EST) (2) and develop mutual problem-solving guidelines for school-age obesity prevention.

## Methods

### Design

The PAR approach was used in this study. It emphasizes developing collaboration among researchers and stakeholders to seek solutions (Stringer, 2014). EST was used as its conceptual framework for the purpose of understanding the complex factors influencing school-age obesity and guiding interventions at the individual, family, and school levels.

### The study area and participants

The purposive sampling technique was used to select the key participants from 36 selected public primary schools in Nakhon Nayok Province, Thailand, which were schools with the highest statistics for childhood obesity in Thailand and willing to participate in the study. The stakeholder analysis indicated names of 55 stakeholders as key informants as shown in Table 1.

**Table 1.** The stakeholders in the study

Focus group (8 groups, 48 people)	In-depth interview (7 interviews)
**Students (n = 24)** Group 1 – Normal weight students in Grades 1–3 (6 students) Group 2 – Normal weight students in Grades 4–5 (6 students) Group 3 – Overweight students in Grades 1–3 (6 students) Group 4 – Overweight students in Grades 4–5 (6 students) *(Consideration was given to the students’ grade levels to ensure that students were independent in their expressions of opinion and to reduce the age differences among the students)*	**School administrator (n = 1)** School administrator (1 people)
**Parents/Guardians (n = 12)** Group 5 – Parents/Guardians of normal weight students (6 people) Group 6 – Parents/Guardians of overweight students (6 people)	**School lunch cooks (n = 5)** –Store No. 1 (2 people) –Store No. 2 (3 people)
**Teachers (n = 7)** Group 7 – Teachers (7 people) –Home-room teacher in Grades 1-5 (5 people) –Home-room teacher in Grades 6 (2 people)	**School nurse (n = 1)** School nurse (1 people)
**Shop vendors (n = 5)** Group 8 – Vendors in front of the school (5 people)	

### Data collection approaches

Focus group discussions and in-depth interviews took place at school where an audio recorder and field notes were employed. Each focus group discussion and individual in-depth interview lasted an hour or less. The advisory professors were asked to examine content accuracy and completeness in addition to making corrections. Next, three qualified experts consisting of a nutrition therapist, a paediatric nutrition expert, and a paediatrician expert examined content validity. The EST guide facilitated the researcher concerning the factors and served as a framework for the semi-structured and open-ended questions.

Focus Group Guidelines: The guidelines covered the student group (6 items), the parents group (14 items), and the teacher group (15 items), asking about the perception of their children’s obesity, the causes of obesity, types of activities used in solving the problem of obesity in the past, and the people with key roles in decision-making, persons responsible for activities, school and family in preventing obesity, participation and links between school and family, barriers/struggles to participating in the programme to seek solutions and combat low participation for future study. The focus group discussions were conducted in small groups of 5-8 participants, each with children, family members, teachers, and shop vendors at school. Data were collected through eight focus groups (8 times) with 48 people as follows, as shown in Table 1.

In-depth Interview Guidelines: 16 items asked about the parents’ perceptions of their children’s obesity, the causes of obesity, food environment in school, physical activity and exercises, sustainable management and policy and curriculum in school related to the prevention of obesity. In-depth interviews were conducted with the following seven people (7 interviews), as shown in Table 1.

Participant Observation: Participant observation enabled the researcher to view the real problem in the real situation. These data were important in the validation and interpretation of the information provided by the participants. The purpose of the observation was to observe the school environment factors. The observation was also aimed at discovering the progress of knowledge, improvement, changes, and learning.

### Protection of human subjects

Ethical approval was obtained from the Ethical Committee on Research in Human Subjects, Faculty of Public Health, Mahidol University. Approval number MUPH 2018-012.

### Data analysis

Descriptive content analysis was applied for data analysis.

### Research process

**Figure 1.**
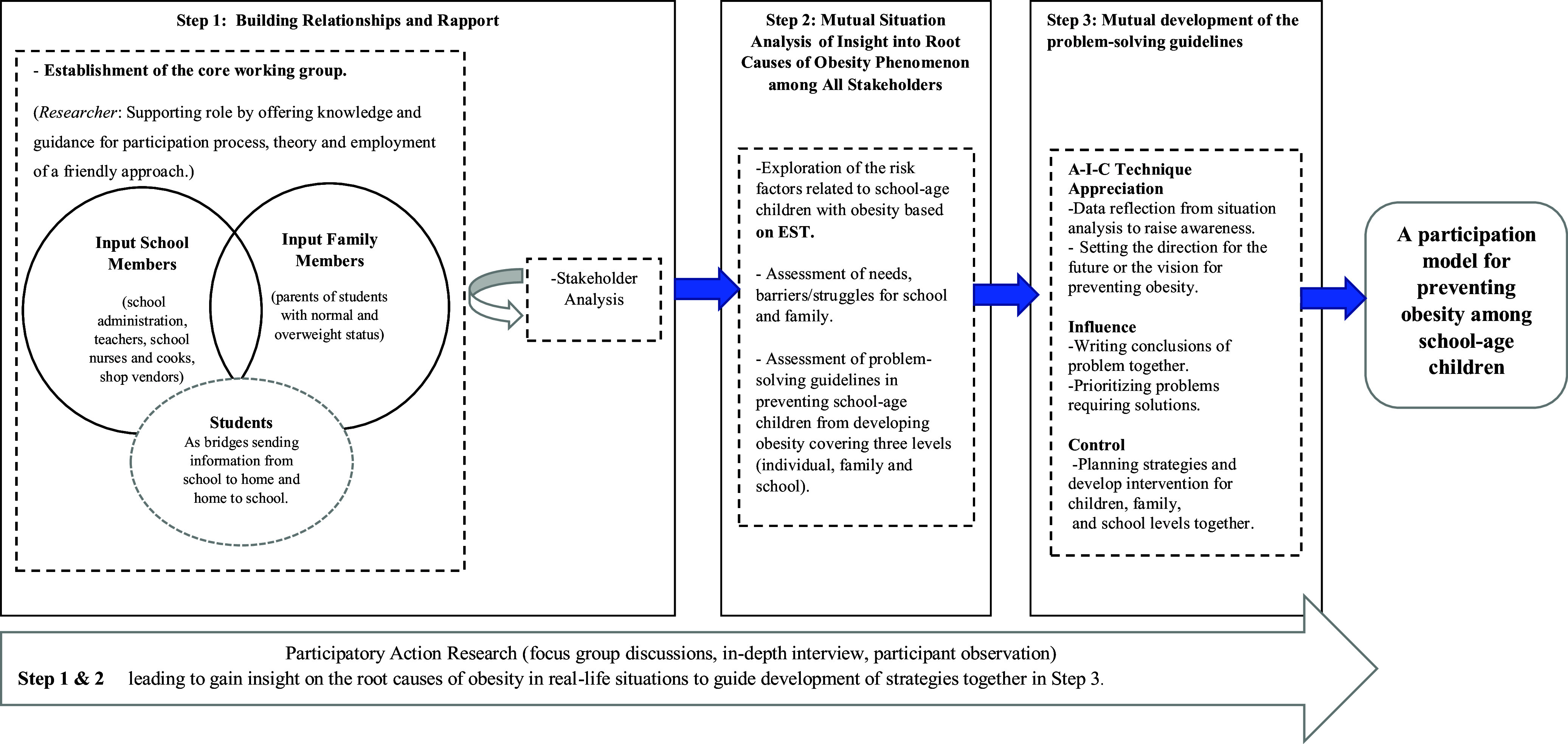
PAR process for school-age children with obesity.

According to Stringer (2014), the participation process was subdivided into three steps: Step I focusing on building relationships and rapport, Step II mutual situation analysis, and Step III mutual development programme among all stakeholders, the details were as follows:

- Step 1: Building relationships and rapport.
  First Meeting – Establishment of the core working group was discussed and decisions were made about candidates for participation. The core working group was an essential step toward an increased understanding of the real problems occurring in the setting and the assignment of responsible people. Nevertheless, the first core working group established was not successful, because the group of people responsible was composed of school executives, rather than willing volunteers. However, adjustments were made by accepting applications from volunteers. After one week, a new core working group was established with willing volunteers who were interested and who had volunteered to serve as representatives to work on preventing school-age children with obesity. The new group consisted of 10 willing volunteers composed of 6 teacher representatives, 3 family representatives and other individuals, including one school director, two teachers from grades 4 and 5, two physical-education teachers, one school nurse and one public health nurse from Ongkharak Hospital to form the core working group. After agreeing on the appointments, the core working group began to identify their roles and proceed with the next step, stakeholder analysis.
  Second Meeting – Stakeholder Analysis (Stringer, 2014): This step involved analysing stakeholders by brainstorming with the core working group. Later, the core working group attempted to perform stakeholder prioritization and identify people potentially affected by the situation of school-age children with obesity. Once every party had agreed to be one of the stakeholder groups, the next step would be to understand the key stakeholders in terms of opinions, needs and likelihood for feelings and responses to the situation of school-age children with obesity by informal interviews. The findings show that the high-power group (high level of power for problem-solving) and high-interest group (high interest in solving this problem) (close management) were the groups requiring full participation and attention in this study. They would be serving as the main caregivers of students, family members (parents, grandparents, and other relatives) and school personnel (physical-education teachers and school administrators).
- Step 2: Mutual situation analysis of insight into root causes of the obesity phenomenon among all stakeholders
  The purpose of this step was essential to gain insight into the root causes of obesity among school-age children in real-life situations in Ongkharak District, Nakhon Nayok Province. As a guide for developing the participation model and planning the strategies for the next step, relevant variables were selected that would cover the factors at each level. Selected variables were based on EST (Bronfenbrenner, 1977), as derived from a previous literature review on school-age children with obesity. The researcher collaborated with representatives from the core working group, and the stakeholders joined in the data collection. The following three methods of qualitative data collection were employed: 1) focus group discussions with eight focus groups (8 times); 2) in-depth interviews with seven people (7 times); and 3) participant observations to conduct the investigation and permit a triangulation approach. After completing the data collection, the researcher analysed all the data by using content analysis and ensuring member checking. The purpose of the researcher was to clarify and discuss the findings within the core working group and all stakeholders to ensure the correctness of the peer-debriefing process and the credibility of the data.
- Step 3: Mutual development of the problem-solving guidelines
  The third meeting employed the AIC technique to provide an opportunity for all the parties to develop the problem-solving guidelines together. The AIC process is composed of the following three main procedures:
  1. **Appreciation**: The researcher collaborated with representatives from the core working group on data reflection with all stakeholders (student, family, and teacher representatives) who were aware of the problem based on real situations in the area. Analysis was focused on setting a direction for the future and creating a vision for preventing the problem of school-age children with obesity. The teachers, parents and children drew pictures of the past and future and displayed their data to all the stakeholders. Pictures that had been drawn from the imaginations of children prompted all stakeholders to set real goals and understand the current needs and thoughts of the children. When all parties had completed their tasks, presentations were made by each party on what it had accomplished.
  2. **Influence**: This involved forming guidelines for developing problem-solving guidelines for the prevention of school-age children with obesity as a joint effort. After agreeing on the final data once conclusions had been drawn, the school and family representatives found the problem of obesity to be complex. Specifically, they wanted to see improvements in health among school-age children after seeing the statistics showing that there were more students with obesity than in the past, while the trend in obesity continued to rise. However, the barriers resulted from parents who were so busy with their work that they had little or no time for their children. The participants prioritized problems by adhering to data from the findings of the previous step and divided the data into the individual, school, and family levels. However, an agreement was made to solve all three levels of obesity problems at the same time without dividing or choosing to solve only one level of problems. The core working group reasoned that the various levels were all interlinked. Therefore, problems should be solved simultaneously to create sustainability. Throughout this process, the researcher worked with the core working group to improve motivation, co-learning, self-awareness, feelings of ownership and self-esteem. Once an agreement had been reached together, the group entered the stages of developing the problem-solving guidelines model to prevent obesity among school-age children and plan strategies for each level based on objectives and goals
  3. **Contro**l: The researcher and core working group worked together to develop problem-solving guidelines, interventions, and operational plans (what, when, who, with and with whom). The researcher played the role of facilitator in the process; no leading questions were used, but necessary suggestions and information were provided. This approach led to a high sense of belonging or ownership among all parties and created a sense of collaborative support in seeking solutions (see Figure 1).

## Results

The results showed four main categories: (1) exploration of the risk factors related to obesity among school-age children based on EST; (2) assessment of the needs for school and family in preventing obesity among school-age children; (3) assessment of the barriers/struggles for school and family in preventing obesity among school-age children; and (4) assessment of the problem-solving guidelines in preventing obesity among school-age children.

1. **Risk Factors Related to School-Age Children with Obesity Based on EST**

The results show that multiple factors contributed to school-age children with obesity at individual level, family level and school level.

**Individual level**: the most important individual factors contributing to the development of obesity, particularly among children who lack the necessary knowledge they need to prevent obesity. Instead, they perceive their obesity as an ‘unrealistic perception about students’ nutritional status’, have consumption behaviours including high-calorie and sweetened beverage intake, less physical activity, and sedentary lifestyles. Moreover, students who have stress and sleep late are at risk for obesity. For example, the normal and overweight students in this study reported.

*‘…we were stressed out from our special classes or having to study all day; this regimen made students feel fatigued and hungry. So, we bought some food to eat before special classes in the evening. Most of the foods we bought were high-calorie foods like fried foods, fried meatballs and fried chicken or sweetened beverages’.* (TSA1)

*‘When I stay up late, I usually feel hungry and open the refrigerator to look for something to eat. I mostly eat bread and Mama (instant ramen noodles)’. (TSA1a)*

Family and school arrange the environments surrounding children that contribute to childhood obesity and EST facilitates greater clarity in understanding the importance of environment based on an EST consisting of four systems: the microsystem, mesosystem, and exosystem, as shown in the model created by the researcher.

**Family level** is the environment that is closest to children and provides direct experiences for children. If the systems in EST are compared, **the microsystem** consists of factors at the family level, or family environmental factors have the most powerful influence over the development and maintenance of children’s eating and exercise habits at home. All the students and parents in this study had the opinion that the relevant family environmental factors were family perceptions, such as, ‘Chubby children are cute’ and ‘Weight will automatically lower with continued growth’. Parenting practices included parents spoiling children and family models. The parents reasoned that ‘*the parents love their children and love to see that their children are happy at mealtime and that the children are happy when they are eating’* (PA1), ‘*parents want children to stay still without bothering anyone or running around’* (PA2) and, ‘*Some parents do not like to eat vegetables, so we make food without vegetables and our kids don’t eat vegetables either’.* (PA3)

However, all the participants in the student, parent, and school groups had the opinion that parenting practices consisted of both positive parenting practices and negative child-rearing behaviours. Negative child-rearing behaviours (no rules at home, spoiling the children, low support for exercise in the family, low access to healthy food at home and eating outside the house frequently) were found to be a cause of obesity, while positive parenting practices (setting rules in the house, supporting exercise, increasing access to healthy foods in the house by cooking healthy food and preparing fruits in the house, cutting or washing fruits and storing fruits in the refrigerator for students to use) were found to be protective factors.

The links between family and school were found to be a risk factor in **the mesosystem** based on EST. Negative links between school and family affected incidences of school-age children with obesity including lack of communication between family and school, lack of participation between family and school and lack of continuity of practice at home.

One parent of an overweight student said, ‘*The school doesn’t send reports about how children are either obese or thin to us at home. They say your child is chubby, but they don’t add anything further. Consequently, I don’t know what to do or how to care for him**/**her. They don’t talk to us about it at all’* (PGT1).

**School level** is an external environment with indirect impacts on the children because children part of their time at school in their studies compared to **the exosystem in EST** such as school environment factors including school health policy and curriculum in school, food environment in school, physical activity, and exercise in school.

The school administrator said, ‘*The curriculum focuses on academic studies in the textbooks. It’s been found that students usually spend over 80% of school hours studying in the classroom in subjects such as math, science and language with only 1-2 hours per week spent studying sports or doing activities outside the classroom, which is considered very little and insufficient’. (SA8) ‘The school used to have policy for organizing an obesity prevention program 5 years ago, but the program no longer exists’. (SA9). ‘The school has no policy about a school lunch project for students, so students are free to choose what they like to eat and the school has no rules and regulations on the types of foods permitted for sale at school’. (SA11)*

The shop vendor said*, ‘Most of the foods I select for sale at school are fried foods, hamburgers, sweetened beverages, sweet snacks and ice cream because the kids like to eat these foods. So, I can sell them at a really good profit. As for fruits and vegetables, I don’t bring them out to sell because they don’t sell. All they do is spoil, rot and result in a loss. It’s about business’. (SV1)*

1. **The Needs for School and Family in Preventing Obesity among School-Age Children**

The school group needed family participation and programme sustainability, while the parent group needed outside help and policy at school regarding obesity prevention, including promotion of healthy foods and menu items made from fruits and vegetables to be sold at school.

1. **The Barriers/Struggles for School and Family in Preventing Obesity among School-age Children**

The most significant barriers for teachers and parents regarding organization or participation in obesity prevention activities were teachers’ heavy workloads. Each teacher taught full-time from morning to evening and the school has an inconsistent policy. Parents lack of time to participate in school activities due to a need to work and earn money to support their families.

1. **Problem-Solving Guidelines in Preventing Obesity in School-Age Children**

**Figure 2.**
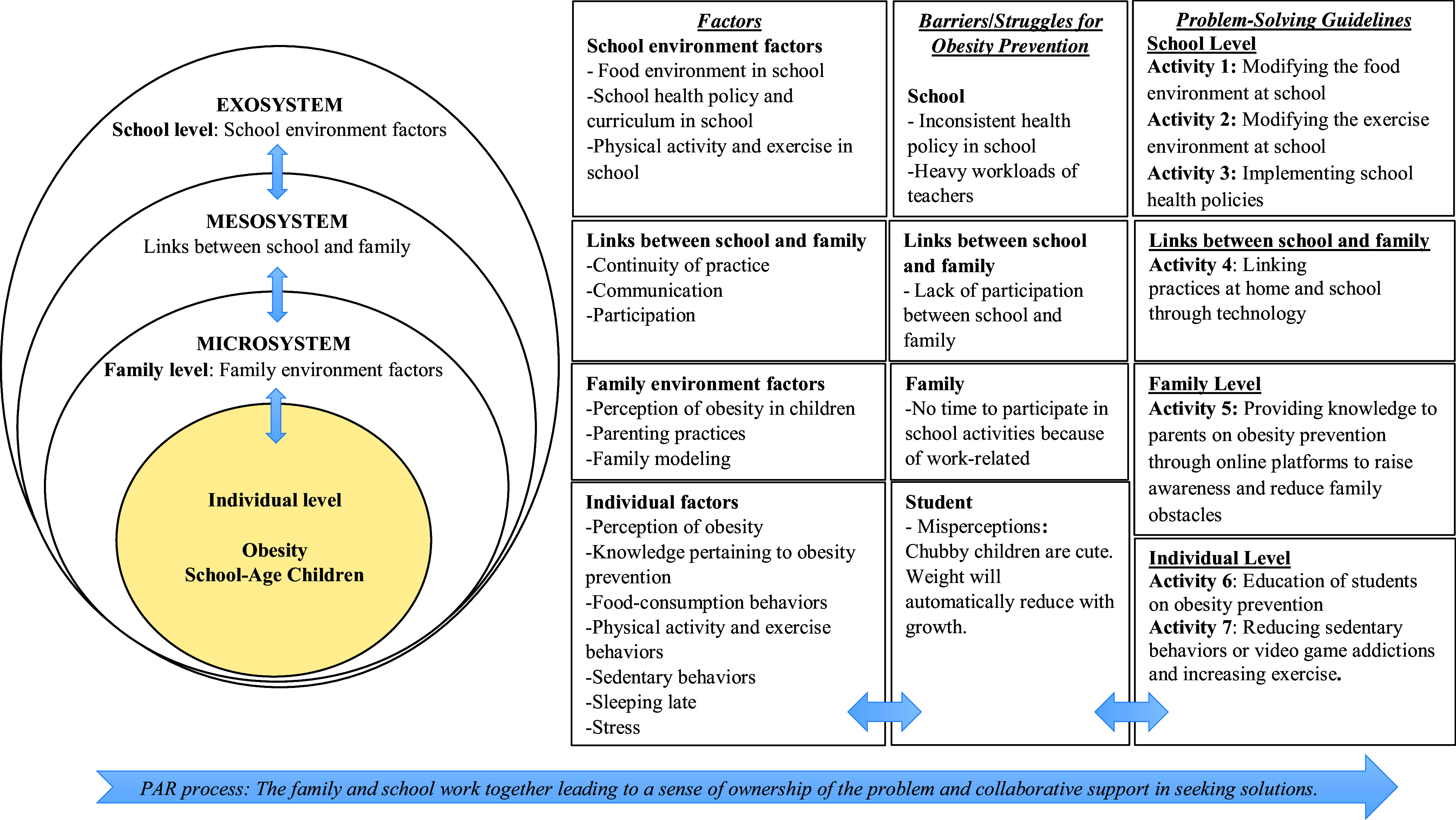
Model for preventing obesity among school-age children.

The results show many guidelines for solving the problem of school-age children with obesity. Once goals and objectives had been set to schedule activities with adherence to risk factors at each level based on the EST, a consensus was reached on the most appropriate interventions for problems. The researcher was able to summarize the conclusions drawn by the core working group working together with the researcher (see Figure 2).

## Discussion

This study applied a qualitative approach. Data were collected from focus group discussions and in-depth interviews for the purposes of finding the risk factors for childhood obesity in school-aged children and seeking problem-solving guidelines for the future. This study adhered to EST from the beginning of the process to the end. Obesity among school-age children was found to involve multiple complex individual and environmental factors (Feeg *et al*., 2014). EST helped build an in-depth understanding about the environmental factors surrounding children at three levels (Zhou and Cheah, 2015; Walker *et al*., 2019). Each level has constant interactions with the others, back and forth amongst the microsystem, mesosystem, and exosystem.

The risk factors for obesity are highly complex, and individual factors might not be the main factors contributing to childhood obesity in school-age children. On the contrary, environmental factors have the greatest influence over school-aged children. The family environmental factors in the microsystem are the most important factors because this environment is closest to children and families foster children’s health care behaviour, not only in terms of food consumption and exercise but also includes building health immunity for children in the future (Pérez Solís *et al*., 2015). The second part is the school environment in the exosystem, an environment that should not be overlooked. School-age children spend part of their time in school, so the fact that children’s school life depends on rules, regulations, teaching curriculum and school policy is unavoidable (Sirikulchayanonta *et al*., 2011; Niemeier *et al*., 2012; Phaitrakoon *et al*., 2014; Syrad *et al*., 2015). Both family and school, therefore, constitute important environmental conditions in preventing childhood obesity in school-age children. EST helped the researcher recognize that, between the microsystem and exosystem, there is another important system linking both of the other systems, and that is the mesosystem.

The greatest need of families and schools in relation to preventing childhood obesity is the need for sustainable continuity. Moreover, previous research has made the decision that having no sustainable solutions to the problem of obesity is a heavy problem being currently faced. The findings of the current study reveal that the core element in building sustainability is links between school and family (Rattanagreethakul *et al*., 2010; Kothandan, 2014; Phaitrakoon *et al*., 2014). This interrelationship is consistent with previous studies showing that obesity prevention among school-age children cannot be achieved by solving only one issue (Hoelscher *et al*., 2015). In other words, continuity and consistency in children’s health care that comes from participation between family and school will lead to successful creation of sustainability.

The barrier that needs to be corrected is the lack of time available for families and schools to participate in activities together. Families have no time, because they were working to make a living, and teachers have limited time because of overwhelming teaching workloads (Hayes *et al*., 2019). The findings suggest the use of technology to help with establishing collaboration between schools and families to enable communication at any time and place. Similarly, previous studies have clearly demonstrated that technology such as websites and the mHealth interactive programmes can engage family participation through the education of the family on weight monitoring and management for overweight children (Lee *et al*., 2018; Verdaguer *et al*., 2018; Yau *et al*., 2022). In addition, participation with children could be successful by examining the changeability and feasibility of programme ideas, prioritizing, and thinking about who to involve and eliminating possible barriers. Children can serve as bridges more firmly linking home and school. The results highlight the fact that perspectives on both the problems and solutions of family members, particularly children, provide an understanding of their problems and stronger ownership of the interventions with participation in development leading to more attractive, relevant, and effective interventions in the future (Anselma *et al*., 2019).

The key success factors in this study were: 1) adaptation of the PAR process and EST as principles for developing participatory processes and 2) discovering the aetiology of obesity in school-age children to find ways to prevent recurrence. This adaptation made operations systematic, while the work done with many sectors was not confusing. In addition, time was neither lost nor wasted, even though the researcher was the only supporter and facilitator.

The study’s limitation was the long time spent in data collection because the teachers and parents had no time, making it difficult to make appointments.

The strengths of the study were the applications of the EST and PAR processes, which provided data with full coverage from the people involved, including students, parents, teachers and school administrators, which made seeking solutions to the problem something every party truly wanted in addition to creating feelings of true ownership over the issue.

## Future directions

For future studies, the findings can be applied to public health nursing work around school health promotion, a policy-driven for school-based participation and environmental support to promote health-promoting school.

## Conclusions

The findings from this study indicate that the risk factors for school-age children with obesity are composed of multiple complex components pertaining to individual and environmental characteristics that can be defined in terms of family and school environments. This study highlights the reality that sustainable prevention of obesity will be difficult if solutions for school and family environmental risk factors at each level are separated, or if only one of the levels is solved, since each factor may affect the other factors at each level. Therefore, school collaboration and strong family participation can enhance sustainability for the prevention of school-age children with obesity. Meanwhile, the students can continue to serve as a bridge between those two parties (the school and the family). Both the PAR and EST can serve as a basis for helping to enhance the participation of all parties and to develop an appropriate context-based programme to prevent non-communicable diseases (NCDs) within the population, especially among school-age children with obesity.
